# First Time Presentation of Graves' Hyperthyroidism With Psychotic Symptoms: A Case Report

**DOI:** 10.1192/bjo.2024.650

**Published:** 2024-08-01

**Authors:** Oluwatobi Ajewole, Victor Doku

**Affiliations:** 1 Oxleas NHS Foundation Trust, London, United Kingdom; 2 King's College, London, United Kingdom

## Abstract

**Aims:**

Graves’ disease, an autoimmune illness, is one of the most common causes of thyrotoxicosis and often presents with classic symptoms of hyperthyroidism. However, patients can rarely present for the first time with psychiatric symptoms, including psychotic and mood symptoms or a combination of both, and there is limited data on the most effective treatment.

**Methods:**

Here, we report the case of a 24-year-old black British female who had no previous psychiatric or medical history, presenting for the first time with one week history of poor sleep, disordered thought, and bizarre and violent behaviour towards family. Collateral history describes her premorbid personality as “anxious and perfectionist”, with the only recent stressors identified being preparations for her best friend's wedding. Her mental state on presentation was remarkable for tangential and circumstantial speech, incongruent affect, and lack of insight into illness. She was admitted to an acute adult ward under Section 2 of the Mental Health Act (MHA) after being “medically cleared” but before the results of her thyroid function tests were available.

She was transferred back to the acute medical ward a day into psychiatric admission, where she was treated medically for thyrotoxicosis and discharged with the support of the Home Treatment Team after an almost complete recovery in her mental state. Initial symptoms recurred two weeks after discharge, culminating in another admission cycle initially to a psychiatric unit under the MHA, where she was treated with oral risperidone and a medical ward for further medical investigations. Her mental state improved significantly again, and she was discharged home to the concerted care of both a community mental health team and follow-up with the endocrinology team. On outpatient psychiatric review a year following discharge, the patient remains stable in her mental state and has achieved a euthyroid state with plans to taper off and withdraw risperidone gradually.

**Results:**

This case shows the importance of a thorough physical health assessment and investigation before making psychiatric management decisions. It also points out the drawback of the divide between physical and mental health services, the impact this has on patient care and experience within the National Health Service, and the mixed success of medical management in controlling psychiatric symptoms.

**Conclusion:**

This case describes the rare presentation and successful management of psychosis induced by thyrotoxicosis in a female patient with Graves’ disease. It highlights the need for prompt, interdisciplinary care to diagnose and safely manage such patients correctly.

